# Study on the pathogenesis of pathophysiological changes of burn systemic infection

**DOI:** 10.1155/S0962935192000553

**Published:** 1992

**Authors:** Li Ao (Ngao), Huang Yuesheng, Yang Zongcheng

**Affiliations:** Burn Institute Southwestern Hospital Third Military Medical College of PLA Sichuan Chongqing 630038 China

## Abstract

The present prospective study showed that incidence of systemic infection in severe burn patients was 30.9%. Toxic shock and multiple organ failure (MOF) developed in all patients with uncontrolled systemic infection. Both morbidity and mortality of MOF were 76.5%. In the infection group, plasma TXB_2_ and TXB_2_/6-keto-PGF_1α_ ratio increased markedly. Their changes were closely correlated with the clinical course and deterioration of systemic infection. Circulatory platelet aggregate ratio decreased significantly, while myocardiac enzyme spectrum greatly increased. Thrombi were observed in visceral tissues from patients dying of systemic infection. These suggested that TXA_2_/PGI_2_. imbalance promoting microaggregate and thrombus formation may be one of the pathogenic effects of toxic shock and MOF in burn patients.


Mediators of Inflammation 1, 371–374 (1992)

### Study on the pathogenesis of pathophysiological changes of burn systemic infection

Li Ao (Ngao),[CA] Huang Yuesheng and Yang Zongcheng

Burn Institute, Southwestern Hospital, Third Military Medical College of PLA, Chongqing, Sichuan 630038, China

[CA] Corresponding Author

**THE present prospective study showed that incidence of systemic infection in severe burn patients was 30.9%. Toxic shock and multiple organ failure (MOF) developed in all patients with uncontrolled systemic infection. Both morbidity and mortality of MOF were 76.5%. In the infection group, plasma $TXB_2$ and $TXB_2$/6-keto-$PGF_{1\alpha}$ ratio increased markedly. Their changes were closely correlated with the clinical course and deterioration of systemic infection. Circulatory platelet aggregate ratio decreased significantly, while myocardiac enzyme spectrum greatly increased. Thrombi were observed in visceral tissues from patients dying of systemic infection. These suggested that $TXA_2/PGI_2$ imbalance promoting microaggregate and thrombus formation may be one of the pathogenic effects of toxic shock and MOF in burn patients.**

**Key words:** Burns, Microaggregate, Prostacyclin, Systemic infection, Thromboxane

### Introduction

It is well known that systemic infection is one of the main causes of multiple organ failure (MOF) and death in severe burn patients.[1] However, most of the previous studies were designed to define how systemic infection occurs, and the mechanisms of the pathophysiological alterations induced by burn systemic infection were seldom investigated. It has been reported[2–4] that thromboxane $A_2(TXA_2)$/prostacyclin in ($PGI_2$) imbalance played important roles in the pathophysiological changes in endotoxic animals. Treatment with $PGI_2$ improved survival of lethal endotoxaemia in dogs.[5] Nevertheless, no reports concerning their roles in the pathophysiological alterations of burn systemic infection have been found. In the present study, we investigated the changes of plasma levels of $TXA_2$ and $PGI_2$ in burn systemic infection patients in order to define the role of $TXA_2$ and $PGI_2$ imbalance in the pathogenesis of pathophysiological alterations of systemic infection in severe burns.

### Materials and Methods

Fifty-five patients admitted to our institute from February 1987 to October 1988 were divided into infection group and non-infection group according to patients complicated with or without systemic infection within 7 days post-burn (PB). In the infection group, there were 17 patients, eleven males and six females. Average patient age was 29.0 ± 3.3 (17–53) years and total burn surface area (TBSA) was 82.5 ± 4.1 (45–98) %. Of the 17 cases, eleven developed septicaemia and six wound sepsis. The non-infection group included 38 patients, 25 males and 13 females, with an average age of 28.6 ± 3.1 (16–58) years and an average TBSA of 51.6 ± 4.1 (30–85) %. The time for occurrence of systemic infection was 1 post-burn day (PBD) in two cases; and 3–7 PBD in 15 cases (88.2%). Among the eleven septicaemia cases, there were 17 positive blood culture with ten strains of different bacteria. *Pseudomonas aeruginosa* was discovered in seven of the 17 (41.2%); both staphylococci and serratia in two (11.8%). Two cases were infected with two, and three cases with three species of bacteria. Five patients developed mixed blood infection with 5 PBD, two cases with two strains, three with three strains of different bacteria.

All patients were given conventional treatment after admission. Blood samples were collected and studied at 0.5, 1, 2, 3, 5 and 7 PBD, respectively. Plasma levels of $TXB_2$, the stable product of $TXA_2$, and 6-keto-$PGF_{1\alpha}$, the stable degradation product of $PGI_2$, were assayed with a radioimmunoassay (RIA) technique. RIA kits were supplied by the General Hospital of PLA, Beijing. Blood samples were drawn with siliconized syringes containing heparin and indomethacin, and then collected in pyrogen-free siliconized glass tubes. Specimens were spun for 15 min at 1500 × $g$ at 4°C and the plasma fraction was extracted twice with ethyl acetate. The extracted supernatant, after drying in a vacuum desiccator, were available for $TXB_2$ and 6-keto-$PGF_{1\alpha}$ determination. RIA was then performed with commercially available standards, trace compounds, and antisera, according to the RIA procedures for $TXB_2$[6] and the description of 6-keto-$PGF_{1\alpha}$ RIA kits of the General Hospital of PLA, Beijing. All assay points were run in

© 1992 Rapid Communications of Oxford Ltd duplicate. The antibodies used to measure $TXB_2$ and 6-keto-$PGF_{1\alpha}$ cross-react less than 0.42% to 0.0025% with other prostaglandins. Circulatory platelet aggregate ratio (CPAR) was determined with the method of Wu and Hoak.[7] The EDTA/formalin solution and EDTA solution were prepared as described by Wu and Hoak.[7] Venous blood (1 ml) was drawn with a siliconized syringe and added into two siliconized glass tubes (0.5 ml for each tube), one containing 2 ml of buffered EDTA/formalin solution and the other buffered EDTA solution only. After thorough mixing, the samples were kept at room temperature (20°C to 25°C) for 15 min and then centrifuged at 150 × *g* for 8 min to obtain platelet rich plasma (PRP). Platelet counts on both PRP samples were determined with light microscopy. Values of circulatory platelet aggregate ratio (CPAR) were obtained by dividing platelet count in EDTA/-formalin PRP by platelet count in EDTA PRP. The myocardial enzyme spectrum (creatinine phosphokinase, CPK; lactate dehydrogenase, LDH; and glutamine oxaloacetic acid transaminase, GOT) was measured by a routine method. Pathomorphological observations of myocardiac, lung, liver and renal tissues immediately taken from seven MOF patients of the infection group who died within 7 PBD were carried out with light microscopy.

*Statistical analysis:* All results were expressed as mean values ±S.E.M. Statistical analysis was performed with analysis of variance.

### Results

*Clinical manifestations:* All patients in the infection group showed obvious toxic symptoms such as high or low body temperature, altered mental status, tachypnoea, tachycardia, ileus, leukocytosis with left shift or leukopenia during the period of infection. With administration of effective antibiotics in four cases (three with septicaemia and one with wound sepsis) the inrection was controlled 2 to 3 days post-infection. In the remaining 13 patients toxic shock or/and multiple organ failure (MOF) emerged subsequently. All the 13 patients succumbed to toxic shock or/and MOF, and ten (76.9%) of them died within 7 PBD. Both the incidence of MOF and mortality of burn systemic infection in this study were 76.5%.

*Plasma levels of $TXB_2$, 6-keto-$PGF_{1\alpha}$ (6-k-P) and $TXB_2$/6-k-P ratio:* The plasma levels of $TXB_2$ in the infection group increased significantly by 12 h post-burn but then gradually decreased during the next 1.5 days (by day 2). They markedly increased again during 3 to 7 PBD. There was no significant difference in the plasma levels of 6-k-P between the two groups. Plasma levels of the $TXB_2$/6-k-P ratio in the infection group, elevated initially at 12 h and during 3 to 7 PBD, showed significant differences compared with those found in the non-infection group (Fig. 1).

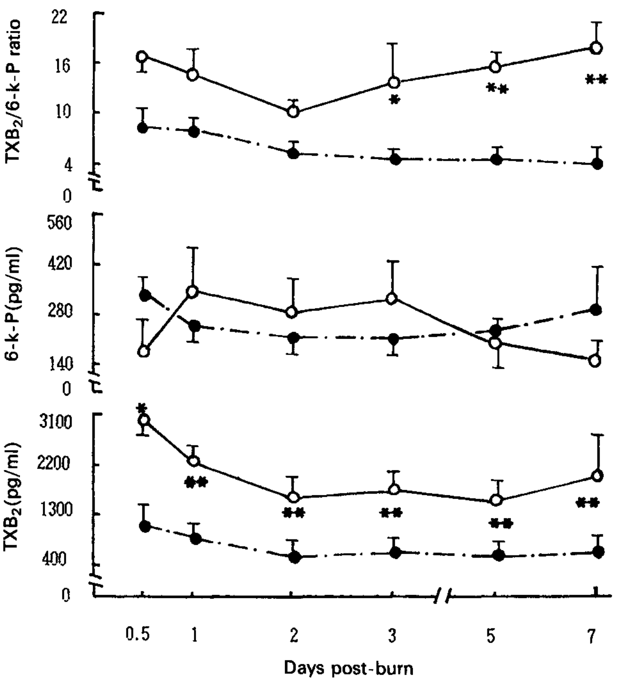

FIG. 1. Dynamic changes in plasma levels of $TXB_2$, 6-keto-$PGF_{1\alpha}$ (6-k-P) and $TXB_2$/6-k-P ratio in infection (—) and non-infection (-·-) groups of severe burn patients. *$p < 0.05$, ***$p < 0.01$.

The circulatory platelet aggregate ratio (CPAR) in the infection group, as shown in Fig. 2, showed no significant difference compared with that in the non-infection group within 1 PBD. After PBD 2 its level remained low in the infection group, but gradually recovered in the non-infection group. The difference between the two groups became significant.

Levels of plasma $TXB_2$ and CPAR in 13 MOF patients of the infection group are shown in Table

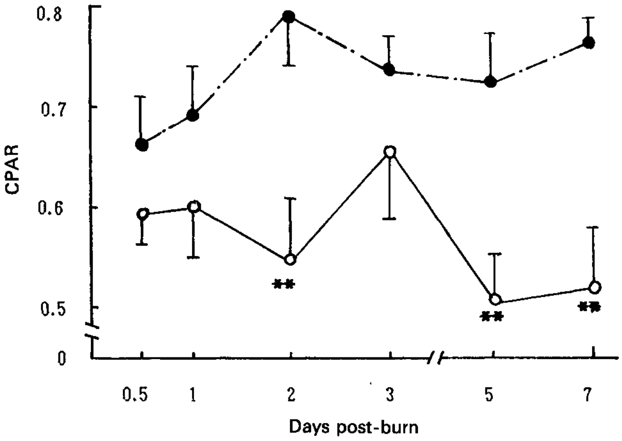

FIG. 2. Changes of circulatory platelet aggregate ratio (CPAR) in infection (——) and non-infection (-·-) groups of severe burn patients. **$p < 0.01$.

**Table 1.** Results of $TXB_2$ and CPAR in 13 MOF patients of the infection group mean ± S.E.M.

| | Before infection | During infection | During MOF |
|---|---|---|---|
| $TXB_2$ (pg/ml) | 2007.1 ± 269.6 | 2715.8 ± 361.3 | 3443.5 ± 266.5 |
| CPAR | 0.62 ± 0.05 | 0.40 ± 0.04 | 0.32 ± 0.04 |

1. $TXB_2$ was higher during the MOF stage than during the infection. In contrast, levels of CPAR further declined when MOF occurred. The level of CPK increased in both groups post-burn. However, in the non-infection group it remained significantly higher and manifested a greater tendency to increase than in the non-infection patients. Levels of LDH and GOT showed similar changes (Fig. 3).

Pathomorphologically, cloudy swelling and fragmentation of myocardial fibres, interstitial oedema and haemorrhage in myocardial tissues were observed. Congestion, oedema, haemorrhage and thrombosis were the main pathomorphological alterations found in the lung tissues. The common findings in renal tissues included ischaemia, swelling, collapse or ischaemic and necrotic changes in the glomeruli, dilatation of tubules containing heme or hyaline casts and thrombosis in the microvasculature. Cloudy swelling, fatty metamorphosis and necrosis were demonstrated in hepatic tissues.

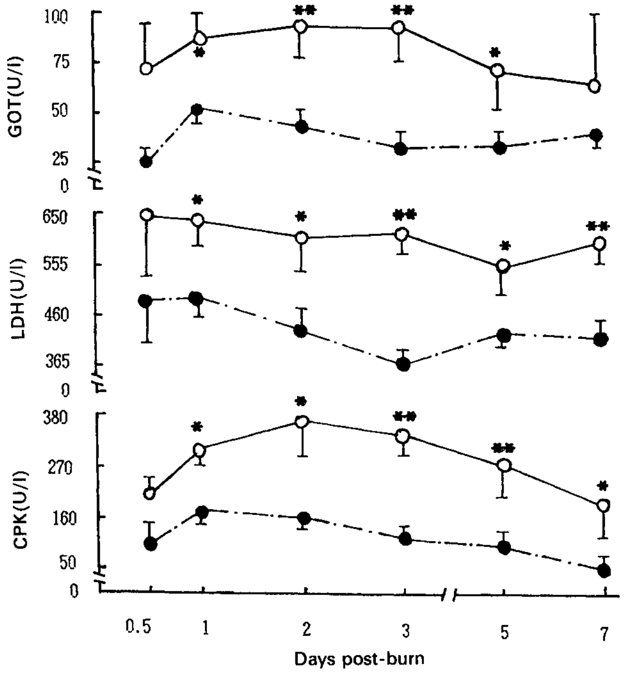

FIG. 3. Changes in levels of myocardial enzyme spectrum (CPK, LDH and GOT) in infection (——) and non-infection (–·–) patients with severe burns. *$p < 0.05$, **$p < 0.01$.

### Discussion

The findings of this present prospective clinical study revealed that the incidence of systemic infection in severe burn patients was 30.9%. Except for four cases, which were controlled by administering antibiotics, 13 of the 17 patients in the infection group developed toxic shock and MOF, and subsequently died of MOF. As is well known, circulatory microaggregate formation is one of the major pathogenic features of toxic shock and MOF.[8,9] Wang and his colleagues[10] found that during the early stage of endotoxic shock, there were microaggregates mainly consisting of platelets in microvasculatures of lung, liver and renal tissues, and the microaggregates, as stated by the authors, played an important role in the genesis of visceral dysfunctions. During the late stage of endotoxic shock, the microaggregates gradually evolved into disseminated intravascular coagulation, which was one of the main causes of death due to deterioration of shock and/or MOF caused by systemic infection. However, the pathogenesis of shock and MOF caused by systemic infection in burn patients is not yet clarified.

It is known that $TXA_2$ is the most potent platelet aggregator known and an extremely strong vasoconstrictor. Conversely, $PGI_2$ is a strong vasodilator and antiplatelet aggregator. The precise balance of these two mediators is an important determinant of the levels of vascular tone, platelet function and many other cellular homeostatic functions.[4] Many previous studies have demonstrated that the imbalance of $TXA_2$ and $PGI_2$ played important roles in the pathophysiological alterations of toxic shock such as pulmonary hypertension, cardiac reduction and hypotension in non-burnt patients.[4,11,12] Treatment with a $TXA_2$ inhibitor showed beneficial effects in endotoxic shock in rats.[13] The results of this study demonstrated that plasma levels of $TXA_2$ and the $TXA_2/PGI_2$ ratio in the infection group were significantly higher than those in the non-infection group. Further analysis of their dynamic changes revealed that the changes of plasma $TXA_2/PGI_2$ ratio within 7 PBD exhibited two peaks of interest. The first peak occurred within 1 PBD and the second during 3 to 7 PBD. As reported before,[14] the first peak might be correlated with the genesis and development of burn shock. Combining the time at which the $TXA_2/PGI_2$ ratio altered with the clinical manifestations and the results of blood bacterial culture of the 17 patients with infection, it was found that 15 of the 17 infection cases developed systemic infection during 3 to 7 PBD, of which 13 cases died of toxic shock and/or MOF during days 3 to 7 of the post-burn period. Our previous studies[14,15] confirmed that $TXA_2$ promoted microaggregate formation and played important roles in the genesis and development of post-burn shock and MOF. The present study also demonstrated that the level of CPRA was lower during infection than before emergence of infection, and further decreased when MOF occurred. In contrast, plasma levels of $TXA_2$ in MOF cases in the infection group increased further. These results substantiated that the second peak of the $TXA_2/PGI_2$ ratio coincided with the development of toxic shock and/or MOF, and the deterioration of the general conditions of the patients with infection. Thus it is proposed that the imbalance between $TXA_2$ and $PGI_2$ may be one of the factors of the pathophysiological changes (shock and MOF) in severe burn patients complicated with systemic infection.

According to the findings of this present study, the promoting action of microaggregates and thrombi by imbalance of $TXA_2$ and $PGI_2$ was the important pathogenesis of pathophysiological alterations in burn infection patients. As stated above, microaggregate formation is one of the important factors leading to toxic shock and/or MOF in the infected victims in non-burn cases. We also observed microaggregates and thrombi in burn patients with MOF.[1] Microaggregates were also observed in the visceral microvasculatures of patients who died of toxic shock.[16] It has been confirmed that $TXA_2$ is a potent platelet proaggregator.[17] The results of this study showed that circulatory platelet aggregate ratio (CPAR) was significantly decreased within 7 PBD and showed further decreases during 5 to 7 PBD, suggesting the increased formation of circulatory platelet microaggregates in burn patients suffering from systemic infection. Furthermore, pathomorphological observations revealed thrombi in the microvasculatures of the main visceral tissues from patients of the infection group in this study. It is well known that microaggregates and thrombi can cause haemorrheological and haemodynamic disturbances leading to tissue and visceral damage via machanical obstruction of microvasculatures and release of chemical mediators.[18] Therefore, the results of this study suggested that microaggregates and thrombi may also be the basis of the pathophysiological changes initiated by systemic infection in severe burn patients. Levels of $TXA_2$ and the $TXA_2/PGI_2$ ratio in the infection group of this study markedly increased and the second peak occurred before the level of CPAR further declined suggesting that imbalance of $TXA_2$ and $PGI_2$ played an important role in the formation of microaggregates and thrombi in the infection group. Levels of CPK, LDH and GOT in the infection group increased continually, reflecting tissue and visceral ischaemia and damage due to haemodynamical and haemorrheological disturbances, and augmentation of microvascular permeability induced by microaggregates and thrombi promoted by increased $TXA_2/PGI_2$ ratio. Therefore, this study substantiated that the imbalance between $TXA_2$ and $PGI_2$ may be one of the factors leading to the pathophysiological alterations of systemic infection in patients with severe burns.

### References

1. Huang, YS, Li A, Yang ZC. Clinical studies on postburn multiple organ failure: its aetiological factors and monitoring. *Burns* 1992; **18**: 26–29.
2. Smith ME, Gunther R, Gee M, Flynn J, Demling RH. Leukoctyes, platelets, and thromboxane A2 in endotoxin-induced lung injury. *Surgery* 1981; **90**: 102–107.
3. Hales CA, Peterson SM, Kong D, Miller M, Watkins WD. Role of thromboxane and prostacyclin in pulmonary vasomotor changes after endotoxin in dogs. *J Clin Invest* 1981; **68**: 497–505.
4. Carmona RH, Tsao C, Trunkey DD. The role of prostacyclin and thromboxane in sepsis and septic shock. *Arch Surg* 1984; **119**: 189–192.
5. Krausz MM, Utsunomiya T, Feuerstein G, Wolfe JHHN, Shepro D, Hechtman HB. Prostacyclin reversal of lethal endotoxemia in dogs. *J Clin Invest* 1981; **67**: 1118–1125.
6. Li ZJ, Yang MF, Hao XH, Fang YX, Song JY. Radioimmunoassay for thromboxane B2 in human plasma. *Med J Chin PLA* 1985; **10**: 35–37.
7. Wu KK, Hoak JC. A new method for the quantitative detection of platelet aggregate in patients with arterial insufficiency. *Lancet* 1974; **iv**: 924–926.
8. Luo ZY, Yu JL, Luo H, *et al.* Study on the pathogenesis of endotoxic shock. *Bull Hunan Med Coll* 1983; **8**: 1–9.
9. Luo ZY, You JL, Kong GY, Wu YJ, Jin LM. Experimental study of the mechanism of endotoxic shock—The role of microaggregation formation in the endotoxic shock. *Bull Hunan Med Coll* 1982; **7**: 27–33.
10. Wang FX, Wang J, Luo ZY. An experimental study on the mechanism of endotoxic shock (EM studies of microaggregates in the microvasculatures of viscerae). *Bull Hunan Med Coll* 1984; **9**: 122–130.
11. Reines HD, Halushka PPV, Cook JA, Wise WC, Rambo W. Plasma thromboxane concentrations are raised in patients dying with septic shock. *Lancet* 1982; **ii**: 174–175.
12. Carrico CJ, Meakins JL, Marshall JC, Fry D, Maier RV. Multiple-organ-failure syndrome. *Arch Surg* 1986; **121**: 196–208.
13. Wise WC, Cook JA, Halushka P, Knapp DR. Protective effects of thromboxane synthetase inhibitor in rats in endotoxic shock. *Circ Res* 1980; **46**: 854–859.
14. Huang YS, Li A, Yang ZC. Roles of thromboxane and its inhibitor anisodamine in burn shock. *Burns* 1990; **16**: 249–253.
15. Huang YS, Li A, Yang ZC. A prospective clinical study on the pathogenesis of multiple organ failure in severely burned patients. *Burns* 1992; **18**: 30–34.
16. Zhu XX, Wu YY. Infectious shock and multiple organ failure. *Chin J Crit Care* 1986; **6**: 9–12.
17. Bunting S, Moncada S, Vane JR. The prostacyclin-thromboxane A2 imbalance: pathophysiological and therapeutic implications. *Br Med Bull* 1983; **39**: 271–276.
18. Huang YS, Li A, Yang C. Effect of thromboxane and prostacyclin imbalance on hemodynamics and hemorrheology in severe burn patients. *J Med Coll PLA* 1991; **6**: 299–303.

**Received 15 June 1992; accepted in revised form 20 August 1992**
